# Hepatocyte Nuclear Factor 3β Plays a Suppressive Role in Colorectal Cancer Progression

**DOI:** 10.3389/fonc.2019.01096

**Published:** 2019-10-22

**Authors:** Juan Wang, Hao Lu, Wei Wang, Nanxin Zheng, Yi Wang, Zhiqian Hu, Gang Ji

**Affiliations:** ^1^State Key Laboratory of Cancer Biology, Department of Digestive Surgery, National Clinical Research Center for Digestive Diseases and Xijing Hospital of Digestive Diseases, Fourth Military Medical University, Xi'an, China; ^2^Department of General Surgery, Chang Zheng Hospital, Second Military Medical University, Shanghai, China; ^3^State Key Laboratory of Cancer Biology, Department of Pharmaceutical and Pharmacy Administration, School of Pharmacy, Fourth Military Medical University, Xi'an, China; ^4^Department of Colorectal Surgery, Changhai Hospital, Second Military Medical University, Shanghai, China

**Keywords:** hepatocyte nuclear factor 3β, colorectal cancer, JAK-STAT signaling, tumor suppressor, prognosis

## Abstract

**Background and Objective:** Hepatocyte nuclear factor 3β (HNF3β) is a key transcription factor in the development of the gastrointestinal tract. However, only few studies have examined its' expression, function and potential clinical significance in colorectal cancer tumorigenesis and progression.

**Methods:** HNF3β expression in colorectal cancer tissue samples of 174 patients was assessed by immunohistochemistry. The results were analyzed with respect to patients' clinicopathological characteristics and survival. Following the *in vitro* cell transfection, MTT, wound healing, and Transwell assays were used to test cell proliferation, migration, and invasion, respectively. Western blot was used to examine IL6, JAK1, and STAT3 protein expression. The potential for tumor formation was evaluated using a mouse xenograft model.

**Results:** HNF3β expression was lower in colon cancer tissue compared to normal tissue and correlated with UICC clinical stage (*P* = 0.001), depth of invasion (*P* = 0.004), regional lymph node metastasis (*P* = 0.007), distant metastasis (*P* = 0.048), and poor survival (*P* < 0.001) in patients with colorectal cancer. Furthermore, HNF3β overexpression impeded proliferation, migration and invasion of SW480 cells via JAK-STAT3 signaling *in vitro*. Moreso, HNF3β overexpression showed a significant growth inhibition of subcutaneous xenograft tumors *in vivo*.

**Conclusions:** The results show that HNF3β acts as a suppressor of colorectal cancer progression and decreased HNF3 β expression is closely related to the poor prognosis. Thus, HNF3β may be a potential molecular target for inhibition of colorectal cancer cells and development of new anti-tumor therapies.

## Introduction

Although some progress has been made in the management of colorectal cancer based on early diagnosis, radical surgery, radiotherapy, and neoadjuvant chemotherapy, recurrence and metastases remain a major cancer management issue which has yet to be solved (1, 2). Therefore, there is an urgent need to explore and define potential molecular biomarkers for early diagnosis, as well as tumor recurrence and metastasis. However, regulatory mechanisms underlying colorectal cancer occurrence and recurrence or metastasis remain to be determined, as few prognostic biomarkers have been identified.

Hepatocyte nuclear factor 3β (HNF3β), is a key member of the hepatocyte nuclear factor (HNF) family with an important role in embryogenesis (3). Indeed, it has been shown that HNF3 transcription factors, especially HNF3α/FOXA1, HNF3β/FOXA2, and HNF3γ/FOXA3, are involved in the development and differentiation of the gastrointestinal tract (4). HNF3β mRNA is expressed during embryogenesis, and is detected during gastrulation in the anterior primitive streak and node with subsequent expression in the gut (5–7). It has been shown that HNF3β deficiency is lethal in the embryonic stage due to failed node and notochord development (8). Moreover, HNF3β is important for postnatal development due to its role in the regulation of normal bile acid and lipid homeostasis (9). Many studies have shown that HNF3β suppression is closely related to tumorigenesis, progression, and metastasis of several different types of cancer including hepatocellular carcinoma, lung cancer, pancreatic cancer, gastric cancer, and thyroid cancer (10–14). However, the role of HNF3β in colorectal cancer development and progression remains to be determined and thus we have decided to examine its role in colorectal cancer patients as well as in the colorectal cancer model *in vivo* and *in vitro*.

## Materials and Methods

### Patients and Tissue Samples

The use of human tissues was approved by Research Ethics Committee of the Fourth Military Medical University and each patient signed the informed consent for using their tissues in the study. All procedures involving human participants were performed in accordance with the 1964 Helsinki Declaration and its later amendments or comparable ethical standards.

Eight inflammation tissues, 14 low-grade and 18 high-grade intraepithelial neoplasia tissues, 174 primary colon cancer tissues, as well as six normal colon tissues were obtained from surgical resections at the Department of Gastrointestinal Surgery of Xijing Hospital, Fourth Military Medical University from October 2010 to November 2011. None of these patients received neoadjuvant chemotherapy or radiotherapy before surgery and all received standard chemotherapy after surgery.

Tumor tissues were histologically examined by two independent experienced pathologists at the Department of Pathology, Xijing Hospital, Fourth Military Medical University. Tumor staging was performed according to the Union for International Cancer Control (UICC) staging system. The following clinicopathological parameters of patients and their tumors were obtained, including: age, gender, histological type and differentiation status, T status, lymph node and distant metastasis, and UICC stage (Table 1). Complete follow-up of patients for least 5 years for survival analysis was also available.

**Table 1:** HNF3β protein expression and clinicopathological characteristics of 174 CRC patients.

			**HNF3β protein expression**, ***n*** **(%)**				
		**PT, *n***	**–**	**+**	**++**	**+++**	***P***
Gender	Male	82	11 (13.41)	32 (39.02)	35 (42.68)	4 (4.88)	0.673^a^
	Female	92	14 (15.22)	41 (44.57)	31 (33.70)	6 (6.52)	
Age, y	≤60	95	14 (14.74)	44 (46.32)	33 (34.74)	4 (4.21)	0.495^a^
	>60	79	11 (13.92)	29 (36.71)	33 (41.77)	6 (7.59)	
Histological type	AC	154	21 (13.64)	63 (40.91)	61 (39.61)	9 (5.84)	0.601^a^
	MAC	20	4 (20)	10 (50)	5 (25)	1 (5)	
Differentiations	Well + moderate	103	14 (13.59)	46 (44.66)	34 (33.01)	9 (8.74)	0.107^a^
	Poor	71	11 (15.49)	27 (38.03)	32 (45.07)	1 (1.41)	
Tumor location	Colon	109	19 (17.43)	46 (42.20)	39 (35.78)	5 (4.59)	0.400^a^
	Rectum	65	6 (9.23)	27 (41.54)	27 (41.54)	5 (7.69)	
UICC stage	0	5	0	0	3 (60)	2 (40)	0.001^b^
	I	24	1 (4.17)	9 (37.5)	13 (54.16)	1 (4.17)	
	II	49	6 (12.24)	15 (30.61)	24 (48.98)	4 (8.16)	
	III	87	15 (17.24)	43 (49.42)	26 (29.89)	3 (3.45)	
	IV	9	3 (33.33)	6 (66.67)	0	0	
T status	Tis+1	12	0	3 (25)	7 (58.33)	2 (16.67)	0.004^b^
	T2	33	2 (6.06)	15 (45.45)	14 (42.42)	2 (6.06)	
	T3	95	16 (16.84)	39 (41.05)	36 (37.89)	4 (4.21)	
	T4	34	7 (20.59)	16 (47.06)	9 (26.47)	2 (5.88)	
N status	0	82	8 (9.76)	27 (32.93)	40 (48.78)	7 (8.54)	0.007^a^
	1 + 2 + 3	92	17 (18.48)	46 (50)	26 (28.26)	3 (3.26)	
M status	0	165	22 (13.33)	67 (40.61)	66 (40)	10 (6.06)	0.048^a^
	1	9	3 (33.33)	6 (66.67)	0	0	
Patient survival	Alive	75	5 (6.67)	25 (33.33)	37 (49.33)	8 (10.67)	<0.001^a^
	Deceased	99	20 (20.20)	48 (48.48)	29 (29.29)	2 (2.02)	

a Chi-squared test with Yates' correction or Fisher's exact test;

b *Spearman's rank correlation analysis*.

*AC, adenocarcinoma; MAC, mucinous adenocarcinoma; PT, patients*.

Progression-free survival (PFS) was defined as the time from primary surgical resection to first local recurrence or distant metastasis, or the date of the last follow-up. Overall survival (OS) was considered the time from primary surgical resection to death or to the last date of follow-up.

### Immunohistochemical (IHC) Analysis

Formalin-fixed paraffin-embedded tissues for the immunohistochemical analysis of protein expression were cut into 4-μm thick sections, which were subsequently deparaffinized in xylene and dehydrated in descending grades of ethanol. Sections were heated in 0.01 M sodium citrate (pH 6.0) for 15 min in the microwave for antigen retrieval. Endogenous peroxidase activity was blocked using methanol with 0.3% H_2_O_2_ for 10 min. The sections were blocked with 10% normal goat serum in phosphate-buffered saline (PBS) for 1 h at room temperature. The sections were incubated with rabbit monoclonal HNF3β antibody (1:250, Abcam 108422) in PBS at 4°C overnight in a humidified chamber. The next day sections were incubated with rabbit horseradish peroxidase-conjugated secondary antibody (1:500, Nichirei Japan) for 1 h at room temperature. The immunohistochemical reaction was visualized by incubating the sections with 0.1 mg/mL 3,3′-diaminobenzidine tetrahydrochloride (DAB) for 5 min at room temperature. All sections were evaluated by two independent trained pathologist.

### Evaluation of IHC Staining

Results of the IHC staining were scored according to both the percentage and intensity of stained cells. Positive staining was scored 1, 2, 3, or 4 when the percentages of stained cells were 0, 1–25, 26–50, 51–75, and >75%, respectively. Staining intensity was scored as 0, 1, 2, or 3 when colorless, pallide-flavens, yellow, and brown. The final staining score was calculated by multiplying the scores for percentage and intensity. For product scores of nil, 1–4, 5–8, and 9–12, the final staining scores were: −, absent; +, weak; ++, moderate; and +++, strong. Tumors with absent or weak immunostaining were classified as having low target protein expression; tumors with moderate or strong immunostaining were classified as high target protein expression.

### Cell Lines and Cell Culture

Cells of the human colon cancer cell line SW480 were purchased from Shanghai Cell Bank and cultured in McCoy's 5A growth medium (Invitrogen, Carlsbad, USA) supplemented with 10% heat inactivated fetal bovine serum (FBS, Gibco, USA) at 37°C in a humidified atmosphere with 5% CO_2_. Cells of the human 293T cell line were purchased from Shanghai Cell Bank and cultured in Dulbecco's Modified Eagle's medium (DMEM; Invitrogen, Carlsbad, USA) at 37°C in a humidified atmosphere with 5% CO_2_.

### HNF3β Transfection

Full-length human HNF3β clone containing full-length human HNF3β cDNA was purchased from Abcam (UK). HNF3β cDNA was subcloned into the *Sgf* I and *Mlu*I digested fragment of pLVTHM/LENTI-BISVECTOR vector to obtain pLVTHM/LENTI-HNF3β. Recombinant lentivirus containing HNF3β was assembled in 293T cells by cotransfection of pLVTHM/LENTI-HNF3β, with pRsv-REV, pMDlg-pRRE, and pMD2G (Gemma, Germany). Lentiviral particles were harvested 48 h after transfection and purified by ultracentrifugation as lenti-HNF3β. As a control, lentiviruses without the HNF3β gene were generated and designated as lenti-green fluorescent protein (GFP). SW480 cells were transfected with lenti-HNF3β or lenti-GFP using Lipofectamine 2000 (Invitrogen, USA) in accordance with the manufacturer's instructions. Each transfection was performed in triplicates.

### Cell Migration and Invasion Assays

To assess the effect of HNF3β on cell migration and invasion ability a transwell assay was performed using uncoated transwell chambers (Millipore, USA) or chambers coated with Matrigel (BD Bioscience, USA). In brief, the lower transwell chamber was filled with media containing 5% FBS, whereas the upper chamber was filled with 1 × 10^6^ cells suspended with medium containing 1% FBS. After incubation of the cells at 37°C for 24 h, the non-traversed cells on the upper surface of the filter (8 μm) were carefully wiped off with a cotton swab. Cells that migrated through the membrane on the bottom surface of the filter were fixed with methanol and stained with 0.1% crystal violet. Cell migration was assessed by counting cells that had migrated through the membrane at six randomly selected fields under the light microscope (200×, Olympus, Japan). For cell invasion assays, the same protocol was used except that the polycarbonate filter was pre-coated with Matrigel. Each experiment was performed in triplicate.

### Wound Healing Assay

In addition to the transwell migration assay, a wound healing assay was performed to assess the effect of HNF3β on cell motility. Briefly, cells were seeded in 6-well plates and incubated at 37°C for 24 h to form a subconfluent cell monolayer (confluence of 90%). A straight linear wound (1 mm wide) was generated across the middle diameter of each well by gently dragging a sterile pipette tip through the monolayer. Cells were incubated at 37°C for 24 h to migrate across the open wound area. At the end of the incubation, the medium was removed and cells were washed and fixed in methanol. The wound healing process was photographed under a light microscope (Olympus, Japan) at 0, 12, and 24 h. Each experiment was performed in triplicate.

### MTT Assay

The effect of HNF3β on cell proliferation was evaluated using the MTT assay. Briefly, cells were seeded in complete medium overnight in a 96-well plate at a density of 4 × 10^3^ cells per well. On the indicated day of culture, 20 μL of MTT (5.0 mg/mL) were added to each well and incubated for 4 h. MTT was incubated with 200 μL dimethyl sulfoxide per well to resolve the formed formazan crystals. The optical density (OD) was measured at a wavelength of 570 nm on a microplate reader (Bio-Rad, USA) every day for the next 5 days (days 2, 3, 4, 5, and 6). The proliferation rate (%) was calculated using the formula: OD_sample_/OD_control_ × 100%. A cell growth curve was drawn according to the daily mean of OD values. Each experiment was performed in triplicate.

### Western Blot Analysis

Cells were lysed in RIPA buffer (1% w/v Triton X-100, 0.1% w/v SDS, 10 mM Tris-HCl pH 7.5, 150 mM NaCl, 1% sodium deoxycholate) supplemented with protease and phosphatase inhibitors (Roche, Switzerland). Equal amounts of protein (40 μg/lane) were loaded and resolved via 12% SDS-PAGE (50 V, 3 h) and then transferred to a nitrocellulose membrane (Perkin Elmer, USA). Membranes were blocked with 5% non-fat milk in Tris-buffered saline for 1 h at room temperature and incubated overnight serially at 4°C with the following primary antibodies: GAPDH (loading control; ab8245; 1:500; Abcam), HNF3β (ab108422; 1:1,000; Abcam), IL6 (ab6672; 1:500; Abcam), JAK1 (ab47435; 1:500; Abcam), and STAT3 (ab68153; 1:1,000; Abcam). The membranes were incubated with the corresponding horseradish peroxidase-conjugated secondary goat-anti-rabbit (1:5,000; sc-2004, Sigma, USA) and goat-anti-mouse (sc-2005) antibodies (1:5,000; Sigma, USA) at room temperature for 1 h. Finally, the blots were visualized with enhanced chemiluminescence (Amersham Biosciences, USA) in according with the manufacturer's protocol and detected using an Image Quant LAS 4000 Mini Bimolecular Imager (GE Healthcare, USA). Each experiment was performed in triplicate.

### Mouse Xenograft Model

The Animal Ethics Committee of the Fourth Military Medical University approved the tumor formation experiment with mice. All the mice were obtained from the Chinese Academy Shanghai Experimental Center, housed under specific pathogen-free conditions, and maintained in an animal facility in accordance with the guidelines of the Johns Hopkins University Animal Care and Use Committee. Eighteen 5-week-old male BALB-C/nude mice were randomized into three groups (each group consisted of six mice): lenti-HNF3β, lenti-GFP, and normal controls. Subcutaneous tumors were established by injecting SW480 cells (5 × 10^7^) per mouse to the dorsal right flank. The tumor diameter was measured every 2 days upon their appearance. Tumor volume was calculated using the length and width of the tumor. The mice were euthanized 6 weeks after the subcutaneously injection, and the tumors were excised and weighed.

### Statistical Analysis

All statistical analyses were performed using IBM SPSS version 22.0 and GraphPad Prism 5 software. Continuous variables are shown as mean ± standard deviation. Categorical variables are stated as number (percentage). The chi-squared test or Fisher's exact test and unpaired Student's *t*-test, or one-way analysis of variance or Mann–Whitney *U*-tests were used to assess possible correlations between HNF3β in tumor samples and clinicopathological factors, depending on variable type and the data distribution. Survival analysis was performed by the Kaplan–Meier method and differences in survival were compared using the log-rank test. The Cox regression model was performed to test the independent prognostic value. *P* values were two-sided and *P* < 0.05 was considered statistically significant.

## Results

### HNF3β Protein Expression in Colonic Tissue Samples

**Figure 1:**
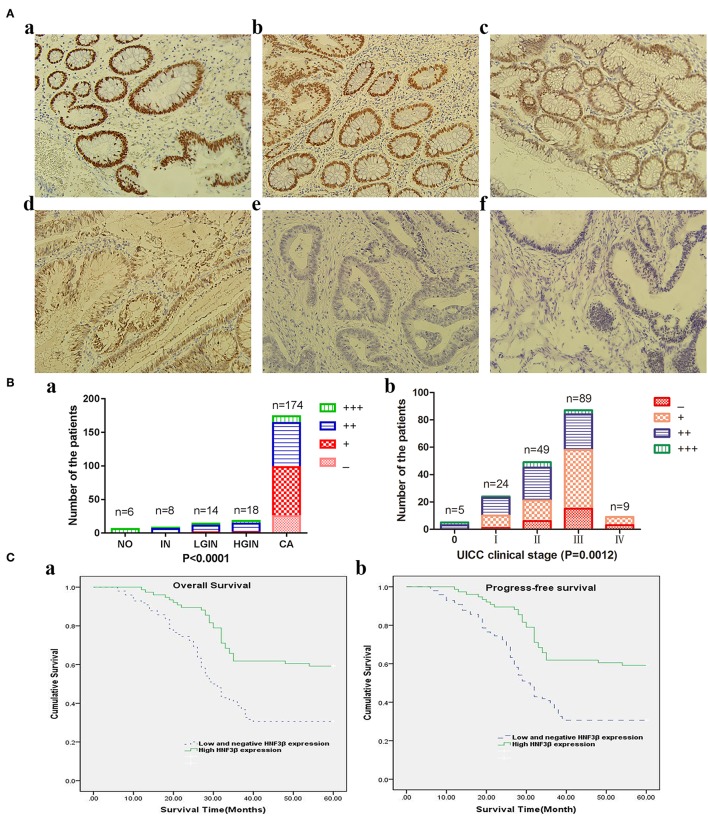
**(A)** Immunohistochemical analyses of HNF3β protein expression in colon cancer and non-cancerous colon tissues. (a) Normal colon tissue; (b) inflammation tissue; (c) low-grade intraepithelial neoplasia tissue; (d) high-grade intraepithelial neoplasia tissue; (e) colon cancer tissue; and (f) mucinous adenocarcinoma tissue. **(B)** HNF3β protein expression in colon cancer and non-cancerous colon tissues. (a) NO, normal colon tissue; IN, inflammation; LGIN, low-grade intraepithelial neoplasia; HGIN, high-grade intraepithelial neoplasia; CA, cancer. (b) HNF3β protein expression in different colon cancer stages. **(C)** Survival rates of patients with low (dashed lines) or high (bold lines) HNF3β protein expression. (a) OS; (b) PFS.

Based on the staining immunoreactivity score, in 14.37% (25/174) of colon tumors no HNF3β protein expression was observed, that is, staining was negative. The remaining tumor samples were positive for HNF3β protein staining, and 41.95% (73/174), 37.93% (66/174), and 5.75% (107/174) were categorized as weak (+), moderate (++), and strong (+++). Taken together, weak or negative HNF3β protein expression was observed in 56.32% (98/174) of colon tumor samples (Figure 1Ae,f), while normal colorectal tissues examined in this study (6 samples) showed strong positive HNF3β staining (Figure 1Aa). Furthermore, all inflamed tissues (8 samples) were also positive for HNF3β staining (Figure 1Ab) and positive HNF3β protein expression was also detected in 92.86% (13/14) of low-grade intraepithelial neoplasias (Figure 1Ac). Furthermore, none of the examined high-grade intraepithelial neoplasias (18 samples) were positive for HNF3β expression (Figure 1Ad). The difference in HNF3β protein expression between the non-cancerous colon mucosal tissues and colon cancer tissues was statistically significant (*P* < 0.0001; Figure 1Ba). Furthermore, low HNF3β expression was detected in 0/5 stage 0, 10/24 (41.67%) stage I, 21/49 (42.86%) stage II, 58/87 (66.67%) stage III, and 9/9 (100%) stage IV patients (Figure 1Ba,b), showing a trend toward decreased HNF3β expression in advanced tumor stages (III and IV; *P* = 0.0012). This observation indicates that HNF3β low expression is associated with tumor progression and therefore HNF3β may have a tumor suppressor role in colon cancer.

### HNF3β Protein Expression and Clinicopathological Characteristics

HNF3β protein expression was associated with the progression of tumors from stage 0 to IV (*P* = 0.001) and with increasing depth of invasion (T classification) from Tis+T1 to T4 (*P* = 0.004; Table 1). Similar trend was observed in the mix population of TCGA colon cancer data sets (Supplementary Figure 1). In addition, low HNF3β expression was more frequently detected in tumors with regional lymph node and distant metastasis (*P* = 0.007 and *P* = 0.048, respectively). No correlation was observed between HNF3β protein expression and other clinicopathological characteristics, including patient's gender (*P* = 0.673), age (*P* = 0.495), tumor histological type (mucinous adenocarcinoma cf. adenocarcinoma, *P* = 0.601), and differentiation status (*P* = 0.107). Collectively these results again suggest that decrease in HNF3β protein expression may have a role in colorectal tumorigenesis and progression.

### Decreased HNF3β Protein Expression Is Closely Related to Poor Prognosis of CRC Patients

During the entire follow-up period, 56.90% (99/174) of the colon cancer patients died and the postoperative median OS time of all recruited patients was 35 months (95% confidence interval [CI] 29.97–40.03). Nine patients had metastases at the time of the surgery. Of 165 remaining patients, 57.58% (95/165) had local recurrence or distant metastasis after surgery and the median PFS time of 30 months (95% CI 13.23–46.77). The 5-year OS rate of 174 patients was 43.10% and the 5-year PFS rate of 165 patients was 42.42%.

The Kaplan–Meier survival analysis and log-rank test revealed that colorectal cancer patients with low tumor HNF3β protein expression tended to have worse 5-year OS and PFS than patients with high tumor HNF3β protein expression (*P* < 0.001, both; Table 2). The postoperative median OS time of patients with low HNF3β expression was 30 months (95% CI 26.77–33.23) while that of patients with high HNF3β expression was 35 months (95% CI 29.97–40.03). The median PFS time of patients with low HNF3β expression in the tumor was 20 months (95% CI 16.45–23.55) while that of patients with high HNF3β tumor expression was 30 months (95% CI 13.23–46.77). The cumulative 5-year OS and PFS of patients with low HNF3β expression was lower than that of patients with high HNF3β expression (OS, 30.6% cf. 59.2%; PFS, 29.2% cf. 57.9%) (Figure 1C).

**Table 2:** Univariate analysis of prognostic parameters affecting 5-year survival of HNF3β in CRC patients, n/N (%).

		**PFS**	***P***	**OS**	***P***
Total		95/165 (57.58)		99/174 (56.90)	
Gender	Male	45/76 (59.21)	0.858	48/82 (58.54)	0.752
	Female	50/89 (56.18)		51/92 (55.43)	
Age, y	≤60	56/90 (62.22)	0.300	57/95 (60)	0.578
	>60	39/75 (52)		42/79 (53.16)	
Histological type	AC	85/148 (57.43)	0.760	87/154 (56.49)	0.868
	MAC	10/17 (58.82)		12/20 (60)	
Differentiation	Well + moderate	52/98 (53.06)	0.199	54/103 (52.43)	0.193
	Poor	43/67 (64.18)		45/71 (63.38)	
Tumor location	Colon	64/101 (63.37)	0.072	68/109 (62.39)	0.060
	Rectum	31/64 (48.44)		31/65 (47.69)	
UICC stage	0	0/5	<0.001	0/5	<0.001
	I	2/24 (8.33)		2/24 (8.33)	
	II	30/49 (61.22)		28/49 (57.14)	
	III	63/87 (72.41)		60/87 (68.97)	
	IV			9/9 (100)	
T status	Tis+1	0/12	<0.001	0/12	<0.001
	T2	13/33 (39.39)		13/33 (39.39)	
	T3	60/90 (66.67)		61/95 (64.21)	
	T4	22/30 (73.33)		25/34 (73.53)	
N status	0	32/78 (41.03)	<0.001	34/82 (41.46)	<0.001
	1 + 2 + 3	63/87 (72.41)		65/92 (70.65)	
M status	0			90/165 (54.55)	<0.001
	1			9/9 (100)	
HNF3β protein	Negative/low	63/89 (70.79)	<0.001	68/98 (69.39)	<0.001
	High	32/76 (42.11)		31/76 (40.79)	

*AC, adenocarcinoma; MAC, mucinous adenocarcinoma; n/N, obituary patients/patients in group; PT, patients*.

Univariate analyses revealed that a low level of HNF3β protein (*P* < 0.001), UICC clinical stages (*P* < 0.001), depth of invasion (T status; *P* < 0.001), positive lymph node invasion (*P* < 0.001), and distant metastasis (*P* < 0.001) were statistically significant predictors for poor OS. Furthermore, HNF3β protein expression (*P* < 0.001), UICC clinical stages (*P* < 0.001), depth of invasion (T status, *P* < 0.001), and lymph node invasion (*P* < 0.001) were associated with poor PFS (Table 2). However, there was no significant correlation between patients' gender, age, histological subtype, or differentiation status with either OS or PFS (Table 2).

In the multivariate analysis, the Cox proportional hazards model revealed that HNF3β expression [hazard ratio (HR) = 0.601, 95% CI, 0.387–0.933, *P* = 0.023], distant metastasis (HR = 5.89, 95% CI, 1.028–33.772, *P* = 0.046), and UICC clinical stage (HR = 3.737, 95% CI, 1.611–8.668, *P* = 0.002) were independent prognostic factors for OS. Finally, HNF3β expression (HR = 0.606, 95% CI, 0.392–0.937, *P* = 0.024), lymph node metastasis (HR = 0.171, 95% CI, 0.032–0.899, *P* = 0.037), and UICC clinical stage (HR = 9.935, 95% CI, 2.214–44.574, *P* = 0.003) were independent prognostic factors for PFS in colon cancer patients (Table 3).

**Table 3:** Multivariate analysis of colorectal cancer patients' OS and PFS.

	**OS**		**PFS**	
	**Adjusted HR (95% CI)**	***P***	**Adjusted HR (95% CI)**	***P***
Lymph node metastasis	–	–	0.171 (0.032–0.899)	0.037
Distant metastasis	5.890 (1.028–33.772)	0.046	–	–
UICC clinical stage	3.737 (1.611–8.668)	0.002	9.935 (2.214–44.574)	0.003
HNF3β expression	0.601 (0.387–0.933)	0.023	0.606 (0.392–0.937)	0.024

Collectively these findings suggest that low HNF3β protein expression in colorectal tumors could be closely related to poor prognosis of colorectal cancer patients.

### HNF3β Overexpression Suppresses the Growth, Migration, and Invasion Ability of SW480 Cells

**Figure 2:**
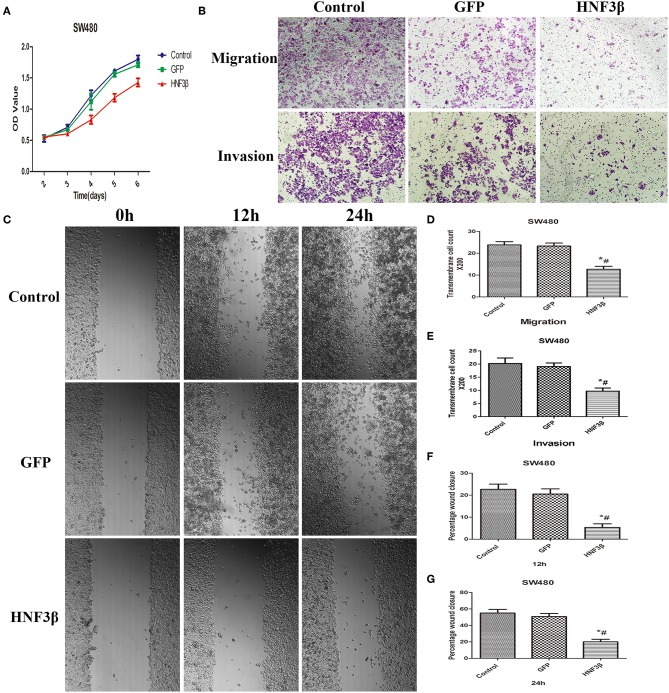
**(A)** MTT assay assessing the proliferative effects of HNF3β overexpression in SW480 cells *P* < 0.05; ^#^control cf. HNF3β; ^*^GFP cf. HNF3β. **(B–G)** Effect of HNF3β overexpression on migration, invasion, and wound healing ability of SW480 cells.

Since we identified a correlation between HNF3β low protein expression in tumor samples and clinicopathological parameters of colon cancer invasion and metastasis, we hypothesized that HNF3β may play a tumor suppressive role in colon cancer progression. By quantitative PCR, we identified HNF3β expression in several colon cell lines and found that it was lowest in SW480 cells (Supplementary Figures 2, 3). Therefore, MTT, transwell migration and invasion, and wound healing assays were performed in SW480 cells to explore the effect of HNF3β on cell growth, migration, and invasion (Figure 2A). Compared with the lenti-GFP transfected and non-transfected cells, the proliferation of the lenti-HNF3β-transfected SW480 cells was significantly decreased from day 3 to day 6 (*P* < 0.05). Furthermore, the migration and invasion abilities were significantly suppressed by HNF3β overexpression in transfected cells, compared with cells transfected with lenti-GFP the non-transfected cells (migration, 12.67 ± 1.37 cf. 23.33 ± 1.37 cf. 23.83 ± 1.47, *P* < 0.0001; invasion, 9.67 ± 1.21 cf. 19.00 ± 1.41 cf. 20.17 ± 2.14, *P* < 0.0001; Figures 2B–G).

The wound-healing assay also showed that cell migration was restrained by HNF3β overexpression compared with lenti-GFP and non-transfected SW480 cells at 12 and 24 h (mean relative migration distance: at 12 h, 5.33 ± 1.63 cf. 20.50 ± 2.35 cf. 22.67 ± 2.34, *P* < 0.0001; at 24 h: 20.00 ± 3.16 cf. 50.83 ± 3.76 cf. 55.00 ± 4.47, *P* < 0.0001; Figures 2D–F). Consistently, these results showed that HNF3β overexpression significantly impeded the proliferation, migration, and invasion ability of SW480 cells.

### HNF3β Overexpression Inhibits the Growth of Subcutaneous Xenograft Tumors in Nude Mice

**Figure 3:**
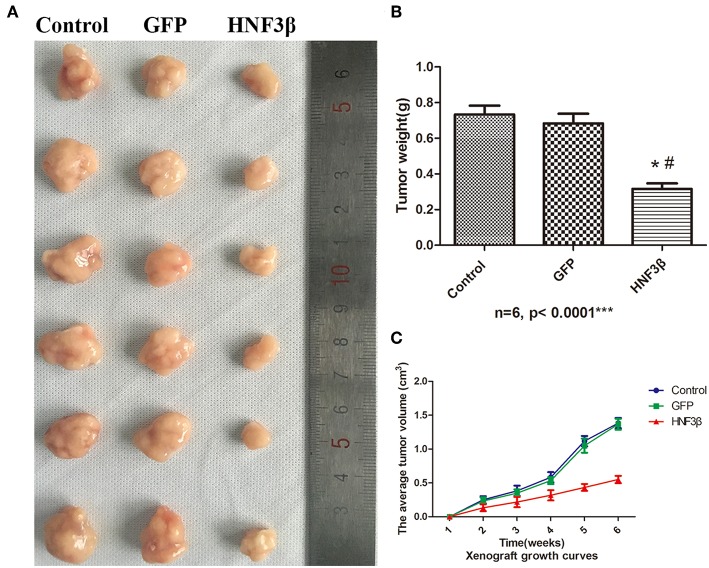
Effect of HNF3β overexpression on xenograft tumor growth. **(A)** The tumors removed from mice. **(B)** Tumor weight. **(C)** The average tumor volume over the time of the experiment. *P* < 0.05; #control cf. HNF3β; ^*^GFP cf. HNF3β.

To examine the potential inhibitory effect of HNF3β overexpression on colon cancer growth *in vivo*, we set up a subcutaneous xenograft tumor model by subcutaneously injecting lenti-HNF3β SW480, lenti-GFP SW480, or SW480 cells in nude mice. The average final tumor volume and weight in the lenti-HNF3β group (0.55 ± 0.05 cm^3^ and 0.32 ± 0.08 g) was significantly suppressed compared with the lenti-GFP group (1.37 ± 0.08 cm^3^ and 0.68 ± 0.13 g) or control group (1.38 ± 0.07 cm^3^ and 0.73 ± 0.12 g; *P* < 0.0001 and *P* < 0.0001; Figure 3). Collectively, these results indicate that HNF3β overexpression in tumor cells may have a suppressive role in the progression of colon cancer.

### HNF3β Overexpression Inhibited IL6, JAK1, and STAT3 Expression in SW480 Cells

**Figure 4:**
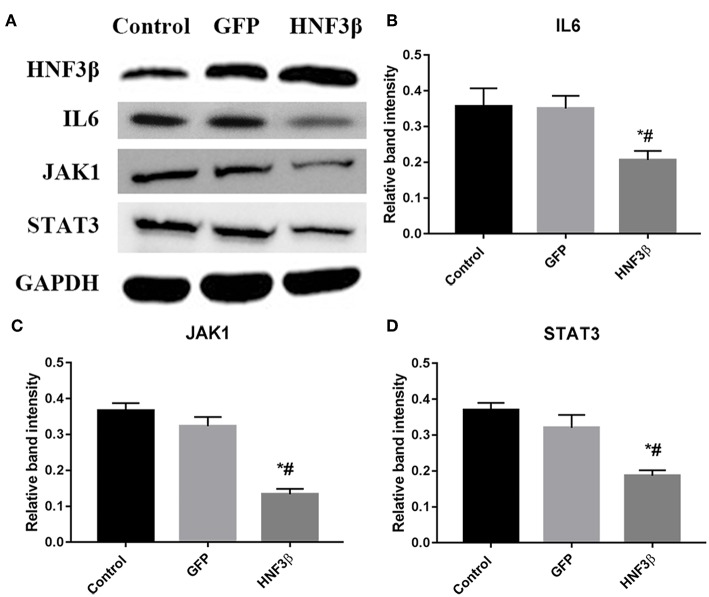
Influence of HNF3β overexpression on protein expression of key JAK/STAT signaling in SW480 cells. **(A)** western blot analysis of IL6, JAK1, and STAT3 protein expression. **(B–D)** relative protein expression of IL6, JAK1, and STAT3 in non-transfected, empty vector transfected, and lenti-HNF3β tranfected SW480 cells *P* < 0.05; ^#^control cf. HNF3β; ^*^GFP cf. HNF3β.

To understand the underlying molecular mechanism through which HNF3β inhibits the growth, migration, and invasion of colon cancer cells, we screened the pathways that HNF3β was probably involved in using western blot. We found that HNF3β overexpression accompanied with decrease in expression of several key genes in JAK-STAT signaling. These included the following: IL6 (control cf. GFP cf. HNF3β, 0.36 ± 0.05 cf. 0.35 ± 0.04 cf. 0.21 ± 0.03, *P* = 0.0237); JAK1 (control cf. GFP cf. HNF3β, 0.37 ± 0.02 cf. 0.32 ± 0.03 cf. 0.13 ± 0.02, *P* = 0.0004); and STAT3 (control cf. GFP cf. HNF3β, 0.37 ± 0.02 cf. 0.32 ± 0.04 cf. 0.19 ± 0.02, *P* = 0.0044; Figure 4). However, no direct interaction between HNF3β and STAT3 was detected by co-immunoprecipitation (Supplementary Figure 5). To explore if HNF3β was involved in interferon γ (IFNγ)-STAT1 signaling so as to affect the STAT3 signal pathway, we have examined the IFNγ expression by quantitative PCR and ELISA. Non-significant change was found in HNF3β transfected cells comparing to the control (Supplementary Figure 4). Analysis of HNF3β involved pathways in STRING database showed that various of molecules correlated to STAT3 signaling, such as AKT1, mTOR, GSK3β, etc., and were likely to interact with HNF3β (Supplementary Figure 6).

Taken together, these findings suggest that HNF3β suppresses the growth, migration, and invasion of colon cancer cells by JAK-STAT3 signaling.

## Discussion

HNF3β has been described in the scientific literature as one of the most important transcription factors involved in gastrointestinal tract differentiation. It has also recently been implicated in tumorigenesis and thus we decided to examine its status in colorectal cancer.

The HNF3 gene family, including HNF3α, HNF3β, and HNF3γ (also known as FOXA1, FOXA2, and FOXA3, respectively), has a highly conserved DNA binding domain, the winged helix domain, which binds to the DNA sequence of the target gene in a monomeric form (15). HNF3β is the founding member of the HNF3 family of transcription factors (16), and its low expression was reported previously in tumor tissues and cell lines. Indeed, over the years accumulating evidence from a number of studies has supported the view that HNF3β genetic alterations or suppression were closely related to the occurrence, progression, or metastasis of several kinds of tumors, including gastric, thyroid, and lung cancer (10, 11, 14). For instance, in the study by Basseres et al. (17), 24.2% (42/173) of non-small cell lung cancers had a HNF3β deletion and in 55.4% (96/173) of these tumors low HNF3β expression was observed. Halmos et al. (10) further confirmed that HNF3β expression in lung cancer cell lines was low compared with normal lung cells. Furthermore, HNF3β expression negatively correlated with the migration ability of lung cancer cells (17, 18), while HNF3β overexpression could induce growth reduction, promote proliferation arrest and apoptosis, and inhibit metastasis of lung cancers (10). Other studies have also shown that HNF3β expression was decreased in papillary thyroid carcinoma and melanoma cell lines (11, 19). HNF3β putatively has a role in the progression of Barrett's esophagus to esophageal adenocarcinoma (20). More recently, inactivation of HNF3β has been implicated in inflammation-mediated liver cancer carcinogenesis (21, 22). However, HNF3β expression and its function in colon cancer has not been systematically investigated.

We analyzed HNF3β expression in the public TCGA database, which consisted of a mixed population including only 12 Asian cases. It showed a non-significant trend of decreasing HNF3β expression with increasing tumor stages, suggesting that more data on HNF3β in East Asian population were required and of great value. Our results show that HNF3β expression was significantly decreased in colon cancer tissues, compared with normal mucous tissue. Furthermore, low HNF3β expression significantly correlated with advanced tumor stage, cancer invasion, regional lymph nodes, and distant metastasis. Colon cancer patients with HNF3β low expression had shorter 5-year OS and PFS compared with those with higher HNF3β expression. Moreover, HNF3β expression was an independent prognostic factor for colon cancer patients. Besides the findings obtained from the IHC analysis of colorectal tumors, we have also shown that forced expression of HNF3β significantly impeded the proliferation, migration, and invasion of colon cancer cells *in vitro* and *in vivo*.

The JAK /STAT signaling pathway is one of the most important that is involved in the control of inflammatory response, cell proliferation, apoptosis, telomerase activity, angiogenesis, tumor invasion, and metastasis (23). Some studies reported a regulatory interaction between STAT3 and HNF3β (24, 25). However, the correlation between HNF3β and STAT3 signal pathway has not been well documented and no related study was conducted in colon cancer. In the present study we have demonstrated that HNF3β suppression of colorectal cancer growth, migration, and invasion may be involved in this pathway and that HNF3β upregulation inhibited the expression of its molecules. Although no direct interaction was found between HNF3β and STAT3, analysis by STRING database showed that HNF3β was likely to regulate STAT3 signaling by a complex, dynamic network via such as AKT1, mTOR and/or GSK3β. In addition, aberrant Akt was found to contribute to maintaining stemness through a NF-kB/IL-6/STAT3 pathway in lung cancer cells (26). The expression of STAT3 can be reduced by mTOR blocking, which is closely related to the invasion and metastasis of liver cancer cells (27). STAT3 is also a target of GSK3β and GSK3β inhibition reduced STAT3 phosphorylation while higher GSK3β expression promoted esophageal squamous cell carcinoma progression through STAT3 *in vitro* and *in vivo* (28). Therefore, HNF3β probably regulates STAT3 signaling via the network including those and more other molecules, and inhibition of this inflammatory signaling pathway may be the mechanism through which HNF3β inhibits the malignant progression of colon cancer. STAT1 signaling that was mainly activated by IFNγ was known to regulate STAT3 signaling (29). However, our data showed that HNF3β was not involved in IFNγ-STAT1 pathway, which implied that the underlying mechanism of how HNF3β is involved in STAT3 signaling remains to be defined in future studies. Finally, gene expression in cell lines is not exactly the same as in tissues due to their different histological origins, microenvironment and tumor heterogeneity, nevertheless, observations obtained from clinical samples are usually more representative of the real conditions *in vivo*. In addition, examining expression of target genes in larger sets of normal tissue specimens would be of great use, however obtaining them is often difficult.

To the best of our knowledge, our study is the first to identify downregulated HNF3β as an independent poor prognostic factor in colon cancer. In addition, in this study we are the first to demonstrate its inhibitory role on malignant behavior of colon cancer *in vitro* and *in vivo* and identified the possible underlying molecular mechanism of its action through modulation of JAK-STAT signaling. Based on these data, we speculate that HNF3β plays a suppressive role in colon cancer tumorigenesis, and that it can be used as a potential new biomarker and therapeutic target in the control of colon cancer progression.

## Data Availability Statement

The datasets generated and analyzed during the present study are available from the corresponding author on reasonable request.

## Ethics Statement

The studies involving human participants were reviewed and approved by Research Ethics Committee of the Fourth Military Medical University. The patients/participants provided their written informed consent to participate in this study. The animal study was reviewed and approved by Animal Ethics Committee of the Fourth Military Medical University.

## Author Contributions

ZH, GJ, and JW conceived and designed the study. JW, HL, and WW performed the experiments. JW wrote the paper. NZ and YW participated in the statistical analysis. WW, ZH, and JW reviewed and edited the manuscript. All authors read and approved the manuscript and agree to be accountable for all aspects of the research in ensuring that the accuracy or integrity of any part of the work are appropriately investigated and resolved.

### Conflict of Interest

The authors declare that the research was conducted in the absence of any commercial or financial relationships that could be construed as a potential conflict of interest.
