# Supplementary material for: Hepatocyte Nuclear Factor 3β Plays a Suppressive Role in Colorectal Cancer Progression
Source: Front Oncol. 2019 Oct 22;9:1096. doi: 10.3389/fonc.2019.01096 (PMC6817462; doi:10.3389/fonc.2019.01096)
Supplement: Supplementary file 1 [file Data_Sheet_1.doc]

**Supplementary materials**

**Materials and Methods**

***RNA Isolation and Quantitative Polymerase Chain Reaction (qPCR) Analyses***

Total RNA was isolated using the TRIzol reagent (Invitrogen, Cergy Pontoise, France) according to the manufacturer’s instructions. cDNA synthesis was performed using the PrimeScript RT reagent kit with gDNA Eraser (Perfect Real Time) (Takara Bio, Shiga, Japan) at 37°C for 15 min and 85°C for 5 s. Real-time PCR reactions were performed using SYBR Premix Ex Taq (Takara, Japan) and the ABI Prism 7500 fast platform (Applied Biosystems, Foster City, CA) with the following conditions: denaturation step, 95°C for 30 s; 40 cycles of denaturation at 95°C for 5 s; annealing at 60°C for 30 s, followed by melting curve analysis. HNF3β was amplified with 5’- GGAGCAGCTACTATGCAGAGC -3’ (forward) and 5’- CGTGTTCATGCCGTTCATCC -3’ (reverse) primers, yielding a PCR product of 83 bp. IFNγ was amplified with 5’- TCGGTAACTGACTTGAATGTCCA-3’ (forward) and 5’- TCGCTTCCCTGTTTTAGCTGC-3’ (reverse) primers, yielding a PCR product of 93 bp. β-Actin used as a reference control and amplified with 5’- ACCAACTGGGACGACATGGAGAAA-3’(forward) and 5 -TAGCACAGCCTGGATAGCAACGTA-3’ (reverse) primers, yielding a PCR product of 192 bp. Relative quantity of HNF3β and IFNγ mRNA was evaluated by the ΔΔCt method and normalized to β-actin. The reproducibility of the measurements was assessed by performing triplicate reactions. Experiments were run and analyzed with the 7500 Fast System Software according to the manufacturer’s recommendations (Applied Biosystems).

***ELISA***

The expression of IFNγ was tested using a enzyme-linked immunosorbent assay (ELISA) kit (OptEIA ELISA Kits,550612, BD) according to the manufacturer’s instructions.

***Co-immunoprecipitation (co-IP) assay***

SW480 cells were transfected with lenti-HNF3β or lenti-GFP. Cells were lysed in RIPA buffer (1% w/v Triton X-100, 0.1% w/v SDS, 10 mM Tris-HCl pH 7.5, 150 mM NaCl, 1% sodium deoxycholate) supplemented with protease and phosphatase inhibitors (Roche, Switzerland). Cell lysates were centrifuged at 10,000×g for 10 min at 4 °C. Next, supernatants were harvested and incubated with the primary antibody anti-HNF3β (Abcam) or rabbit normal IgG for 2 h at 4 C. Then, protein A-agarose (Santa Cruz Biotechnology) was added and incubated overnight at 4 C. The beads were collected by centrifugation at 500×g for 5 min at 4 C and then the samples were resuspended in 20 μL of loading buffer. The relevant proteins were detected by Western blotting using anti-STAT3 (Abcam) and anti-HNF3β.

***Protein‑protein interaction network analysis.***

The protein-protein interaction network of HNF3β was established on STRING database.


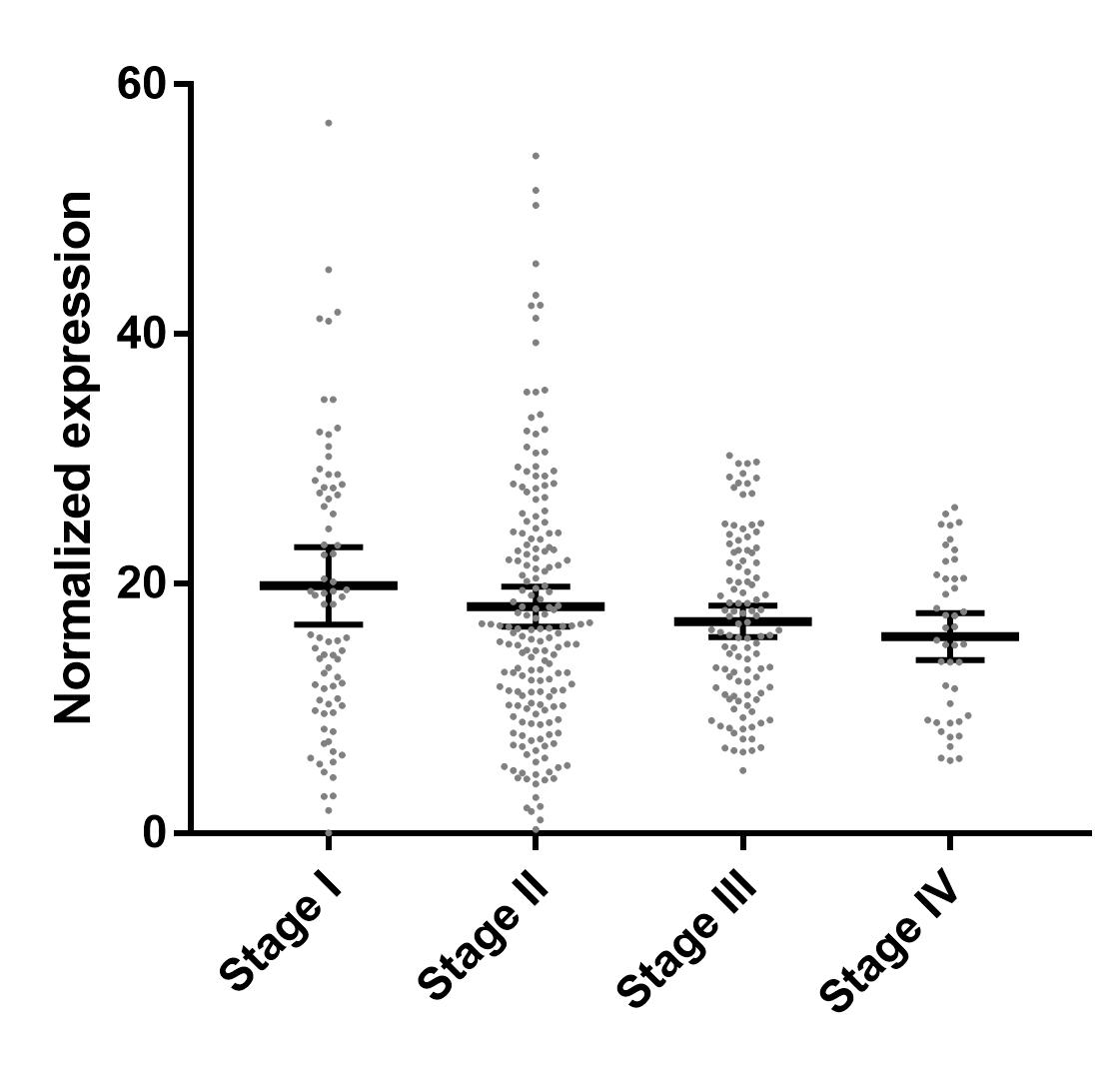


**Supplementary Figure S1.** Analysis of the expression of HNF3β in colon cancer from stage I to IV in TCGA database. Data are shown as means ± standard errors. Kruskal-Wallis test, *P*=0.128.


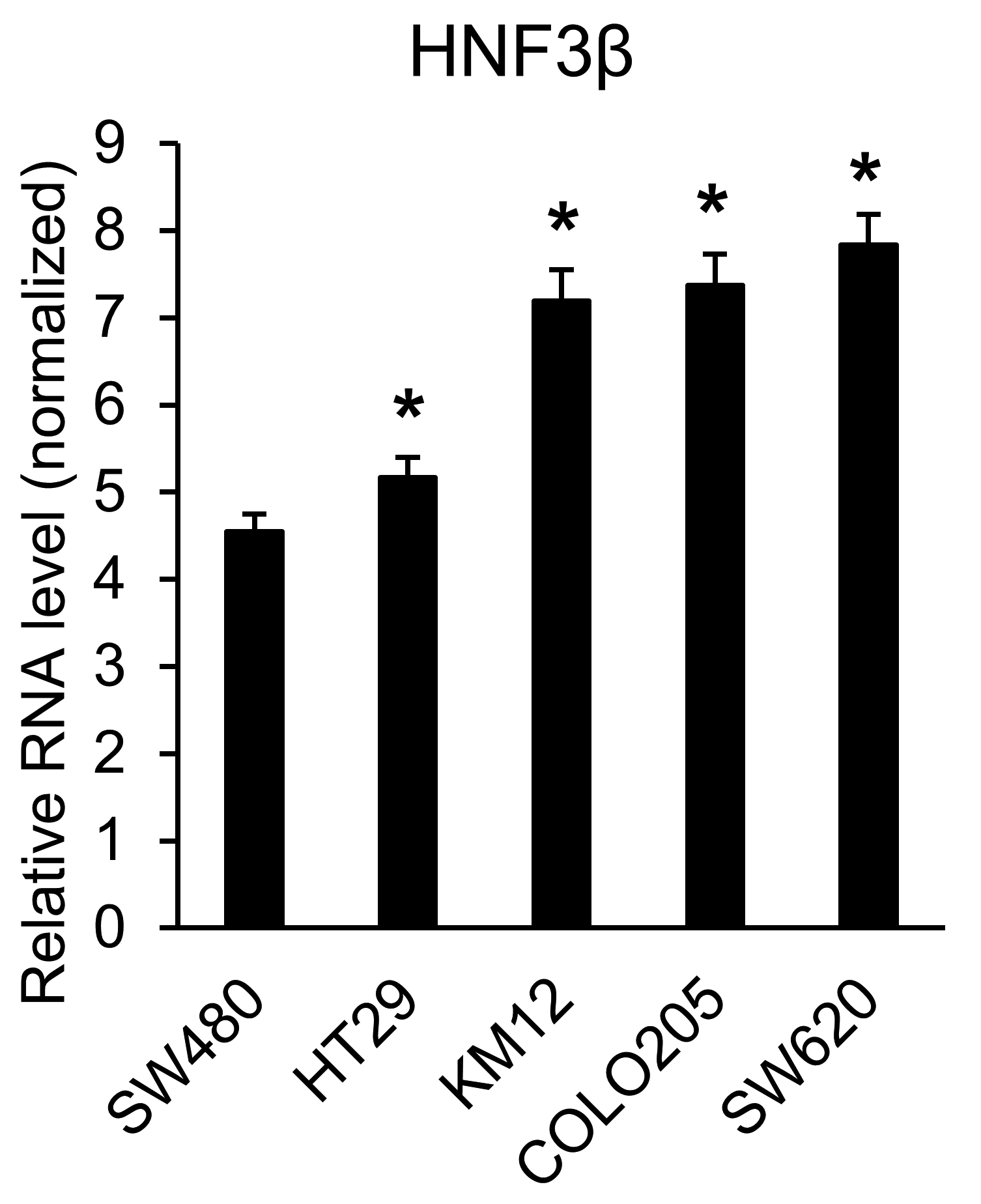


**Supplementary Figure S2.** HNF3β expression in colon cell lines by quantitative PCR. * *P*<0.05 comparing to SW480 cells.


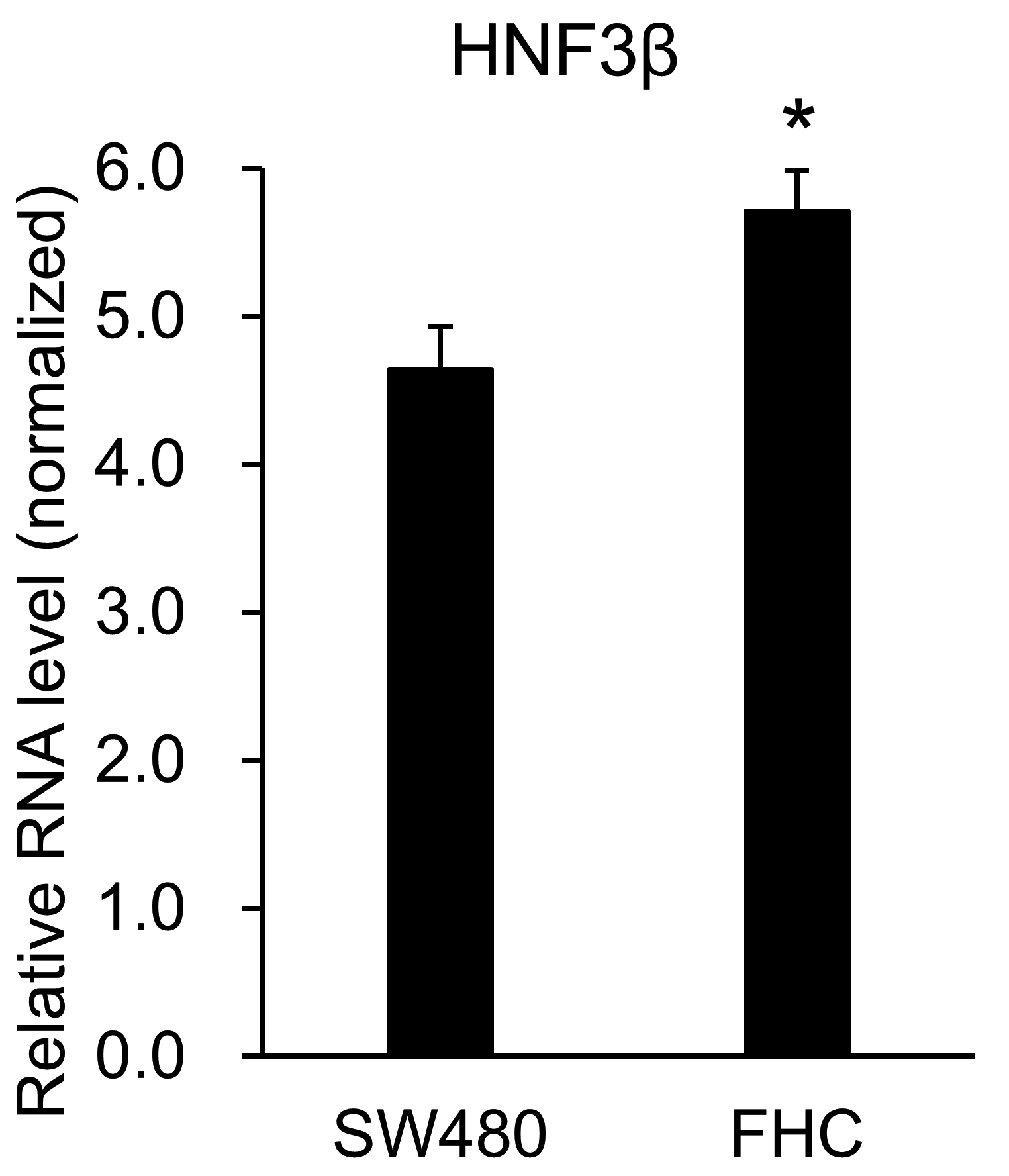


**Supplementary Figure S3.** HNF3β expression in colon normal cell lines FHC compared with colon cancer cell line SW480 by quantitative PCR. * *P*<0.05 comparing to SW480 cells.


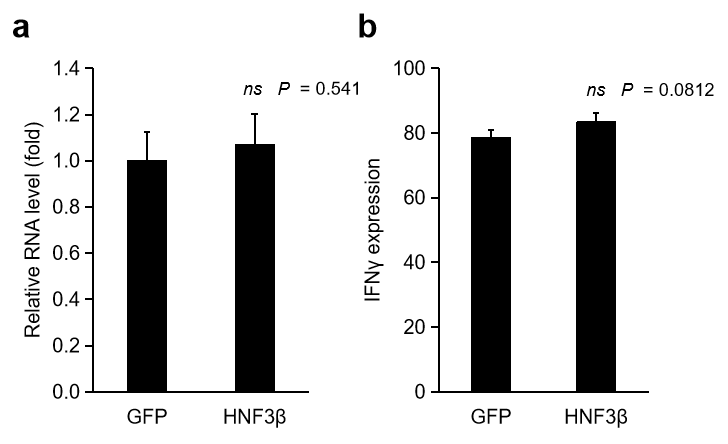


**Supplementary Figure S4.** IFNγ expression in HNF3β transfected cells comparing to the control by quantitative PCR and ELISA. a: Relative expression of IFNγ by quantitative PCR; b: Relative expression of IFNγ by ELISA.


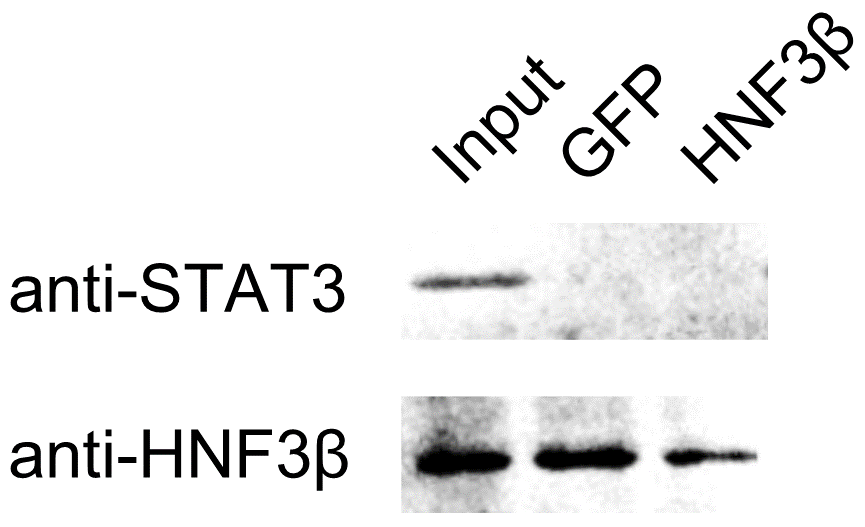


**Supplementary Figure S5.** Co-immunoprecipitation (co-IP) assay of HNF3β and STAT3. Agarose was conjugated with anti-HNF3β antibody. The pulled complex was incubated with anti-STAT3 antibody. Input represents whole cell lysate.


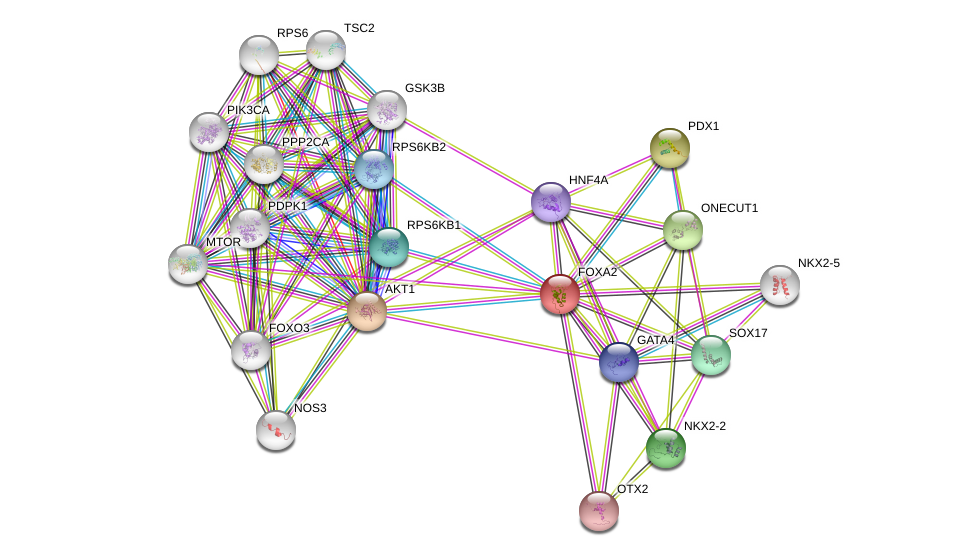


**Supplementary Figure S6.** HNF3β involved molecular network by STRING database.
